# Amide Proton Transfer MRI Signal as a Surrogate Biomarker of Ischemic Stroke Recovery in Patients With Supportive Treatment

**DOI:** 10.3389/fneur.2019.00104

**Published:** 2019-02-22

**Authors:** Lu Yu, Yuhui Chen, Min Chen, Xiaojie Luo, Shanshan Jiang, Yi Zhang, Haibo Chen, Tao Gong, Jinyuan Zhou, Chunmei Li

**Affiliations:** ^1^Department of Radiology, Beijing Hospital, National Center of Gerontology, Beijing, China; ^2^Graduate School of Peking Union Medical College, Beijing, China; ^3^Department of Neurology, Beijing Hospital, National Center of Gerontology, Beijing, China; ^4^Division of MR Research, Department of Radiology, Johns Hopkins University, Baltimore, MD, United States; ^5^Key Laboratory for Biomedical Engineering of Ministry of Education, Center for Brain Imaging Science and Technology, College of Biomedical Engineering and Instrument Science, Zhejiang University, Hangzhou, China

**Keywords:** ischemia, stroke, infarction, acidosis, magnetic resonance imaging, APT

## Abstract

**Background:** Amide proton transfer (APT) MR imaging has shown great potential in the evaluation of stroke severity because of its sensitivity to acid environments. However, this promising MRI technique has not been used to assess treatment efficacy with regard to stroke recovery.

**Purpose:** To assess the therapeutic effect of supportive treatment in ischemic stroke patients using the pH-sensitive APT MRI technique.

**Material and Methods:** Forty-three ischemic stroke patients at an early stage were recruited and scanned with conventional and APT MRI sequences at 3T before treatment. After treatment, 26 patients underwent a follow-up MRI scan (one to three times on different days). The magnetization-transfer-ratio asymmetry at 3.5 ppm, usually called the APT-weighted (APTW) signal, was measured. The APTW signal changes following treatment were analyzed.

**Results:** Baseline APTW signal intensities in the infarcted lesions inversely correlated with baseline stroke severity. Lesion APTW values gradually increased with time in 24 cases (92.3%) with a follow-up MRI scan, showing clinical symptom improvements. Two cases (7.7%) showed further decreased APTW signal in the follow-up scan, accompanied by clinical symptom aggravation. Compared to the baseline, significant APTW signal increases were found for all post-treatment patients (efficacious), whether based on post-treatment or on stroke onset times. The increase in APTW signal in the ischemic stroke lesion after treatment was associated with an improvement in clinical symptoms.

**Conclusion:** The APTW signal would be a useful imaging biomarker by which to assess the therapeutic efficacy of ischemic stroke treatment.

## Introduction

Stroke is one of the leading causes of death, and, due to a lack of adequate treatments, is a cause of extensive concern throughout the world (1). China reports more patients with stroke than anywhere else in the world. It is estimated that approximately two million new cases are diagnosed annually, and approximately one million die from the disease (2). For ischemic stroke, recanalization treatment is an effective way to increase reperfusion rates and to reduce the final infarct size, when administered within the first 4.5 h of symptom onset (3–5). However, due to the time window limitation or a contraindication, many patients fail to accept thrombolysis treatment, usually with supportive treatment. The proper choice of supportive treatment is important for a good outcome. A reliable predictor of treatment response would play an important part in the clinic, especially at the early treatment stage, when it is practical for neurologists to make timely treatment adjustments.

MRI has played an important role in the evaluation of stroke severity and recovery, as well as in the selection of therapy regimens (6, 7). Several different kinds of functional MRI techniques have been used in stroke, for example, diffusion-weighted imaging (DWI) (8–10), perfusion-weighted imaging (PWI) (11), and MR angiography (12). Amide proton transfer (APT) MR imaging (13, 14), a type of chemical exchange saturation transfer (CEST) imaging (15), has shown promise in the detection of a separate pH-based acidosis penumbra in animal stroke models (16–18), even before a diffusion abnormality. Some early human studies have also proved the value of APT MRI in stroke (19–22). However, to our knowledge, no previous studies have been reported about the detection of the pH environment during the process of supportive treatment. In this study, we observed the early treatment effects on ischemic stroke patients using APT-weighted (APTW) (quantified with the magnetization-transfer-ratio asymmetry at 3.5 ppm), and explored the potential of pH-sensitive APTW in the management of ischemic stroke patients at an early stage.

## Materials and Methods

### Subjects

This retrospective study was approved by the Institutional Review Board of Beijing Hospital. Written informed consent was acquired from each subject prior to participation in this study. From April 2014 to March 2015, 61 patients with suspected stroke were enrolled. The inclusion criteria for this study were: diffusion-weighted (DW) images showed areas of restricted diffusion; patients who were not suitable for thrombolysis and only underwent supportive treatment. The supportive treatment that these patients underwent mainly included antiplatelet and anticoagulation therapy, and free radical scavenging. The exclusion criteria for this study were: a history of head trauma; central nervous system infection; other neurologic or psychiatric diseases; or a structural lesion or hydrocephalus on brain magnetic resonance images. The National Institutes of Health Stroke Scale (NIHSS) score was used to measure clinical stroke severity prior to the MR examination. This was performed based on the level of consciousness (LOC), LOC question, commands, best gaze, visual field, facial palsy, motor arm, motor leg, limb ataxia, sensory, best language, dysarthia, and extinction/neglect of the patient by an neurologist: 0–1: normal or near normal, 1–4: mild stroke, 5–15: moderate stroke, 15–20: moderate to severe stroke, and 21–42: severe stroke.

Of the 61 patients, 12 were excluded due to small infarcted regions (<2–3 mm), and six were excluded due to motion artifacts during the MRI scan. As a result, 43 patients (33 men and 10 women; mean age, 64.9 years; range, 44–84 years; Table 1) were included in this study. The first scan was performed prior to treatment for all patients. Some of these patients continued to undergo MRI scanning after treatment, one to three times on different days. The numbers of MRI scans for the 43 patients were as follows: 17 patients with one MRI scan; 13 patients with two MRI scans; eight patients with three MRI scans; and five patients with four MRI scans. In total, 26 patients had follow-up scans. The median onset of symptoms until the first research MRI scan was 3 days (ranging from 6 h to 8 days).

**Table 1:** Basic patient demographic data.

**Patient no**.	**NIHSS at arrival**	**Time since symptom onset**	**Hemisphere**	**Lesion location**	**Territory affected**	**Symptoms**	**Treatment**	**Follow-up MRI**	**Follow-up time (since symptom onset)**
1	6	1 day	R	Parietal and occipital lobe	MCA	Paralysis of left limbs	aspirin, atorvastatin	Y	6, 34 days
2	5	3 days	L	Corona radiata	MCA	Activity disorder of right limbs	aspirin, atorvastatin, low molecular weight heparin	Y	4 days
3	5	2 days	L	Corona radiata	MCA	Paralysis of right limbs and right-sided hypesthesis	aspirin, atorvastatin, low molecular weight heparin	Y	4 days
4	11	2 days	R	Basal ganglia	MCA	Activity disorder of left limbs	aspirin, atorvastatin, low molecular weight heparin	Y	6 days
5	10	19 h	R	Basal ganglia	MCA	Left-sided hypesthesis	aspirin, atorvastatin, low molecular weight heparin	Y	6 days
6	10	2 days	L	Basal ganglia	MCA	Limb weakness and alalia	aspirin, atorvastatin, low molecular weight heparin	Y	4 days
7	2	1 day	R	Corona radiata	MCA	Left limb weakness	aspirin, clopidogrel, atorvastatin	Y	2, 7days
8	3	2 days	L	Parietal lobe	MCA	Right limb weakness	aspirin, clopidogrel, atorvastatin	Y	3, 8, 38 days
9	1	1 day	R	Corona radiata and parietal lobe	MCA	Left limb weakness	aspirin, clopidogrel, atorvastatin	Y	2, 7 days
10	2	10 h	L	Corona radiata and insular lobe	MCA	Right limb weakness	aspirin, atorvastatin	Y	2 days
11	5	20 h	R	Parietal and parietal lobe	MCA	Left limb weakness	aspirin, clopidogrel, atorvastatin	Y	2, 8 days
12	2	6 h	L	Corona radiata	MCA	Right limb weakness and alalia	aspirin, atorvastatin	Y	2, 8 days
13	3	12 h	L	Centrum ovale	MCA	Right limb weakness and dizziness	aspirin, rosuvastatin calcium, low molecular weight heparin	Y	2, 8 days
14	10	1 day	R	Frontal, parietal, temporal and occipital lobe	MCA	Left limb weakness	warfarin	N	
15	9	9 h	R	Frontal, parietal and temporal lobe	MCA	Salivation and alalia	warfarin	N	
16	11	20 h	R	Temporal, occipital, insular lobe and corona radiate	MCA	Left limb weakness	warfarin	N	
17	1	3 days	R	Cerebellar hemisphere	PCA	Dizziness	aspirin, clopidogrel, atorvastatin	N	
18	14	2 days	L	Corona radiata, occipital lobe and insular lobe	MCA	Right limb weakness	clopidogrel, atorvastatin, low molecular weight heparin	N	
19	13	3 days	L	Corona radiata	MCA	Paralysis of right limbs	clopidogrel, atorvastatin, low molecular weight heparin	N	
20	14	1 day	R	Corona radiata	MCA	Paralysis of left limbs	clopidogrel, atorvastatin, low molecular weight heparin	N	
21	6	1 day	L	Posterior limb of internal capsule and thalamus	MCA	Activity disorder of right limbs	aspirin, atorvastatin, low molecular weight heparin	Y	2, 12, 32 days
22	7	3 days	L	Corona radiata and external capsule	MCA	Paralysis of right limbs	aspirin, atorvastatin	Y	10, 32 days
23	6	3 days	L	Corona radiata	MCA	Right limb weakness	aspirin, clopidogrel, atorvastatin	N	
24	5	10 h	L	Corona radiata and insular lobe	MCA	Paralysis of right limbs and alalia	aspirin, atorvastatin	Y	2, 8, 39 days
25	8	1 day	L	Corona radiata	MCA	Alalia	aspirin, atorvastatin, low molecular weight heparin	Y	2, 8 days
26	7	17 h	L	Insular, temporal and frontal lobe	MCA	Right limb weakness and alalia	aspirin, clopidogrel, atorvastatin	Y	3 days
27	4	1 day	L	Insular	MCA	Sensory aphasia and paralysis of right limbs	aspirin, clopidogrel, atorvastatin	Y	2 days
28	3	3 days	R	Frontal and parietal lobe	MCA	Paralysis of left limbs	aspirin, clopidogrel, atorvastatin	Y	4 days
29	1	2 days	L	Temporal and occipital lobe	MCA	Right limb spasm	aspirin, atorvastatin	Y	3 days
30	12	1 day	L	Frontal and parietal lobe	MCA	Paralysis of right limbs and alalia	aspirin, rosuvastatin calcium, low molecular weight heparin	N	
31	2	6 days	L	Parietal lobe	MCA	Paralysis of right limbs and alalia	aspirin, atorvastatin	Y	7, 12, 45 days
32	3	4 days	L	Parietal lobe	MCA	Alalia	aspirin, atorvastatin	Y	7, 8, 31 days
33	8	4 days	R	Temporal and parietal lobe	MCA	Paralysis of left limbs	aspirin, clopidogrel, atorvastatin	Y	7 days
34	1	6 days	L	Temporal and occipital lobe, thalamus and callosum	PCA	Numbness of right limbs	aspirin, atorvastatin	N	
35	10	6 days	L	Frontal and occipital lobe	MCA	Alalia	aspirin, rosuvastatin calcium, low molecular weight heparin	N	
36	2	4 days	L	Corona radiata and basal ganglia	MCA	Right limb weakness	aspirin, clopidogrel, atorvastatin	N	
37	5	6 days	R	Corona radiata, temporal and frontal lobe	MCA	Weakness of both lower limbs and dizziness	aspirin, atorvastatin	Y	11 days
38	10	5 days	L	Frontal, parietal, temporal and occipital lobe	MCA	Paralysis of right limbs	aspirin, rosuvastatin calcium, low molecular weight heparin	N	
39	3	4 days	L	Parietal lobe	MCA	Right limb weakness	aspirin, clopidogrel, atorvastatin	N	
40	5	7 days	L	Corona radiata	MCA	Paralysis of right limbs	aspirin, atorvastatin	N	
41	8	4 days	L	Corona radiata, frontal and occipital lobe	MCA	Paralysis of right limbs	aspirin, clopidogrel, atorvastatin	N	
42	2	4 days	R	Basal ganglia	MCA	Left limb weakness	aspirin, rosuvastatin calcium, low molecular weight heparin	N	
43	1	4 days	R	Frontal lobe	MCA	Left limb weakness	aspirin, atorvastatin	Y	11 days

### MRI Acquisition

All subjects were imaged on a 3 Tesla Philips MRI system (Achieva 3.0T; Philips Medical Systems, Best, The Netherlands), with an eight-channel head coil. The patients were scanned shortly after the neurological assessment. Axial T_2_-weighted, T_1_-weighted, fluid-attenuated inversion recovery (FLAIR), and DWI sequences, were acquired prior to APTW imaging. The APTW imaging slice for each patient was located in the largest hyperintensity area on the DW images. APTW imaging was performed using a 2D single-slice sequence, based on pseudo-continuous wave, off-resonance RF irradiation (saturation duration, 800 ms; power level, 2 μT), and a single-shot, turbo-spin-echo readout. We set the APTW position parameters, including the AP, FH and RL the same as the slice of DWI to further improve registration. The other parameters were as follows: repetition time, 3,000 ms; turbo-spin-echo factor, 54; field of view, 230 mm × 221 mm; matrix, 105 × 100 (reconstructed to be 400 × 400); slice thickness, 6 mm. A multi-offset, multi-acquisition APTW imaging protocol, similar to previous studies (23–25), was used. The 31 offsets were 0, ±0.25, ±0.5,±0.75, ±1 (2), ±1.5 (2), ±2 (2), ±2.5 (2), ±3 (2), ±3.25 (2), ±3.5 (8), ±3.75 (2), ±4 (2), ±4.5, ±5, ±6 ppm (the values in parentheses were the number of acquisitions, which was 1, if not specified). An unsaturated image was acquired for signal normalization. The acquisition time was 3 min 12 s.

### Imaging Processing

The APTW imaging analysis was performed using the Interactive Data Language (ITT Visual Information Solutions, Boulder, CO). To reduce possible motion artifacts during the scanning, the acquired APTW image series for each case was registered to the saturated image at 3.5 ppm, using a rigid-body transformation with three degrees of freedom (26). Then, the z-spectrum (S_sat_/S_0_, in which S_sat_ and S_0_ are the signal intensities with and without selective RF irradiation, respectively, plotted as a function of saturation frequency offset, relative to water) was organized and corrected for the B_0_ field inhomogeneity effect on a voxel-by-voxel basis, as reported before (23–25, 27, 28). CEST imaging is quantified through the magnetization transfer ratio (MTR = 1–S_sat_/S_0_) asymmetry (MTR_asym_) analysis with respect to the water resonance (25):

(1) $$\begin{array}{l} {\text{MTR}}_{\text{asym}}\text{(offset)}=\text{MTR}(\text{+offset})-\text{MTR}(-\text{offset})\\ \;=\left[S_{\text{sat}}\text{(}-\text{offset)-}S_{\text{sat}}\text{(}+\text{offset})\right]/S_{0}\text{.} \end{array}$$

Specifically for APTW imaging at the offset of 3.5 ppm, we have:

(2) $${\text{MTR}}_{\text{asym}}\text{(}3.5\text{ppm})={\text{APTR+MTR'}}_{\text{asym}}\text{(}3.5\text{ppm),}$$

where APTR is the proton transfer ratio for the amide protons associated with mobile cellular proteins and peptides in tissue, and $MT{R^{\prime }}_{\text{asym}}$ consists of various nuclear Overhauser enhancement (NOE) effects of the upfield non-exchangeable protons (such as aliphatic protons) of cellular macromolecules and metabolites (29, 30), including the inherent MTR_asym_ of the solid-phase magnetization transfer effect (25).

**Figure 1:**
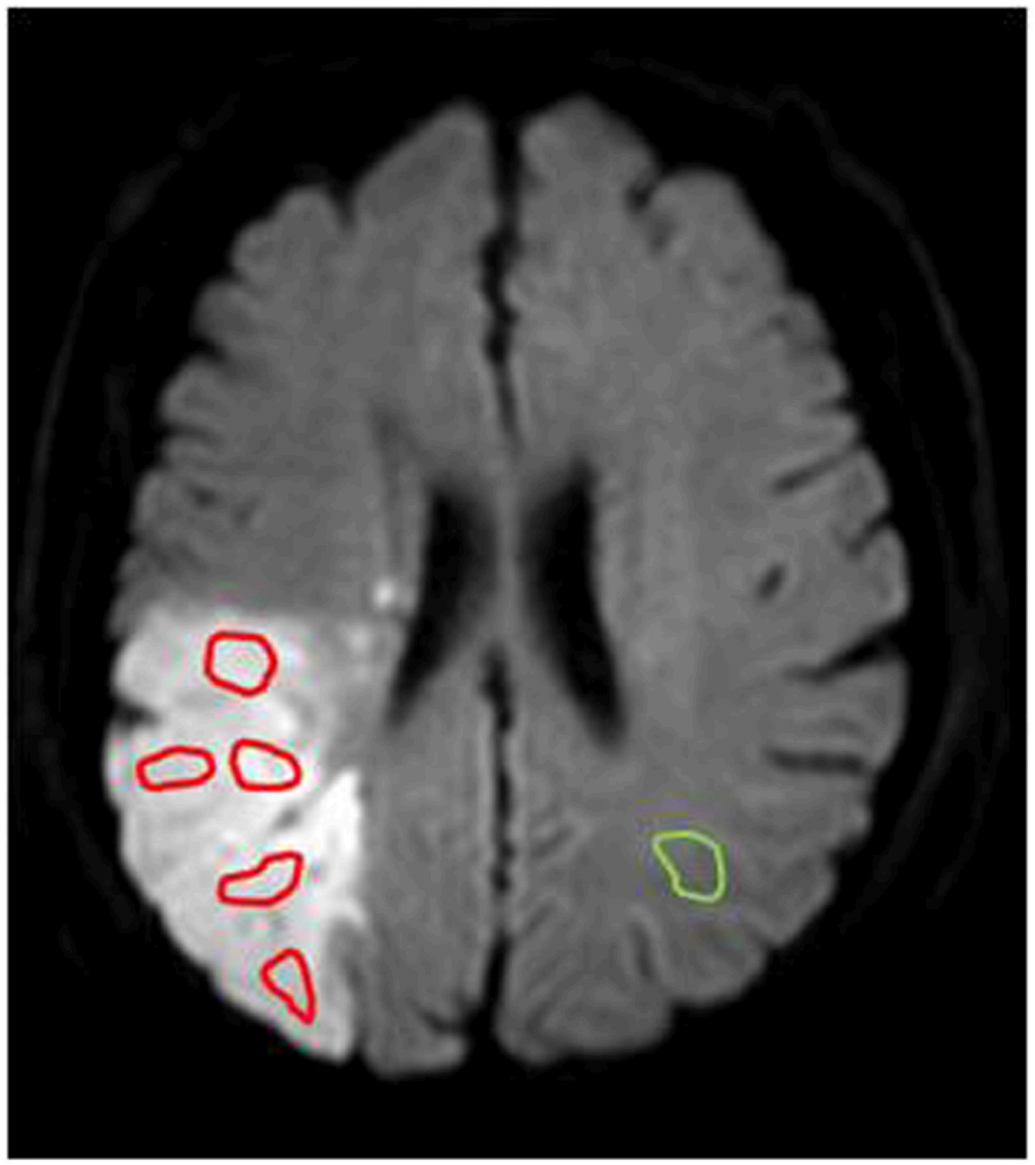
An example of the ROI selection for quantitative APTW image analysis on a DW image. Several ROIs (red, 100~125 pixels each) were selected within the infarcted lesion, and a ROI (green, 100~125 pixels) was selected within the CNAWM for comparison.

For the quantitative APTW image analysis, the DW images co-registered to the saturated image at 3.5 ppm and the corresponding APTW image (26) were used as the anatomical reference to draw regions of interest (ROIs). For each infarct lesion, similar to a few previous reports (27, 28), several small ROIs (Figure 1) were manually selected (100~125 pixels each) on the hyperintense brain regions on DW images by a radiologist who was blinded to patient outcome. The number of ROIs selected depended on the size of the lesion. Theoretically, the lowest APTW value may correspond to the lowest pH in the lesion, a value in which one is interested. Thus, instead of the mean value for each case, the lowest APTW value and the corresponding z-spectrum and MTR_asym_ spectrum data were recorded. The value of contralateral normal-appearing white matter (CNAWM) mirrored the lesion ROIs was also measured for each case. The sulci, hemorrhage, or vessels evident on standard MRI sequences were always avoided. The CNAWM was relatively homogenous, and only one ROI was selected. The APTW difference between an ischemic lesion and the CNAWM (namely, APTW contrast) was also analyzed. In addition, for each infarct lesion, the whole infarct lesion was also manually drawn on the hyperintense brain regions on DW images as ROI.

### Statistical Analysis

All data were analyzed using the statistical package SPSS16.0 (Chicago, IL). Pearson's correlation analysis was performed between the baseline APTW signals and NIHSS at arrival. For patients with more than one scan, the longitudinal signal changes in stroke after treatment were analyzed. We grouped the patients using a time frame similar to that reported in the literature (6). We divided the patients according to post-treatment duration (a time interval between the beginning of treatment and MRI scanning): ≤ 96 h; 4 ~ 7 days; 8 ~ 21 days; and ≥ 22 days. A one-way analysis of variance (ANOVA) test, followed by the least significant difference (LSD) *post-hoc* test, was used to analyze the differences in APTW between pre-treatment and post-treatment with different post-treatment durations. We also divided the patients according to the onset times: ≤ 96 h; 4 ~ 7 days; 8 ~ 21 days; and ≥22 days. APTW MRI signal differences between stroke patients with and without treatment at the same onset time were analyzed by an independent-samples *t*-test. Pearson's correlation analysis was performed for the APTW signals with onset time or post-treatment time. The level of significance was set at *P* < 0.05.

## Results

### Correlation Between Baseline APTW Signals and NIHSS at Arrival

**Figure 2:**
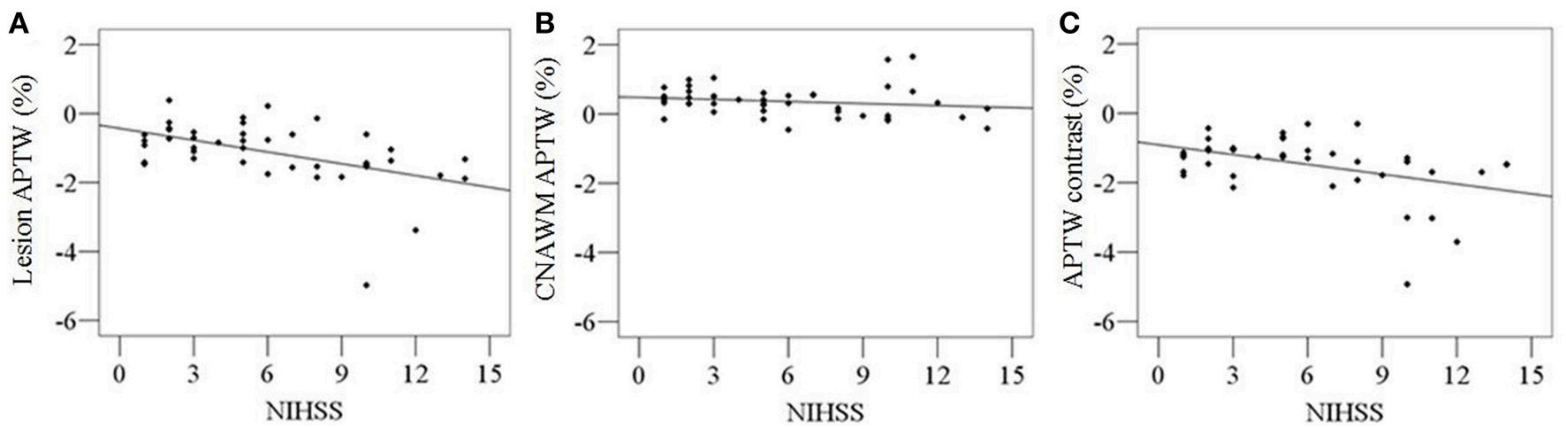
Correlations between pre-treatment APTW signal intensities and NIHSS at arrival. Significant correlations were found between lesion APTW values and NIHSS (*r* = −0.491; *p* = 0.001) **(A)**, as well as APTW contrast values and NIHSS (*r* = −0.425; *p* = 0.004) **(C)**. No significant correlation were found between CNAWM APTW values and NIHSS (*r* = −0.170; *p* = 0.275) **(B)**.

Both lesion APTW values and APTW contrast values had significant correlations with the NIHSS at arrival (Figure 2; *r* = −0.491; *p* = 0.001; *r* = −0.425; *p* = 0.004, respectively), while the correlation between pre-treatment ADC and NIHSS at arrival was not significant (*r* = 0.01, *p* = 0.949). No significant correlation was found between CNAWM APTW values and the NIHSS at arrival (*r* = −0.170; *p* = 0.275).

### Change of MTR_asym_ Spectra After Treatment

**Figure 3:**
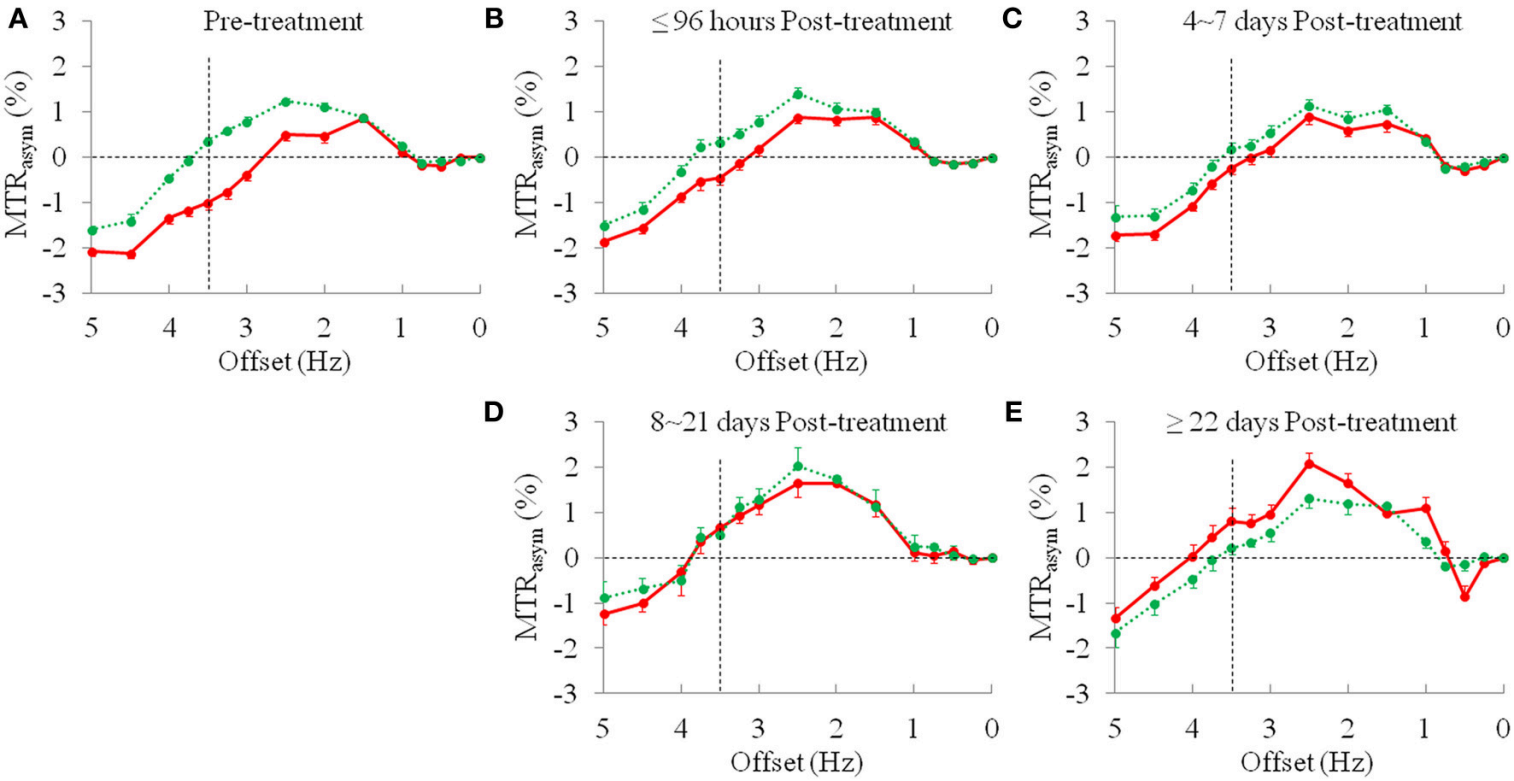
The average MTR_asym_ spectra (mean ± standard error) of the ischemic stroke lesions (red line) and CNAWM (green line). **(A)** Pre-treatment, *n* = 43; **(B)** ≤96 h post-treatment, *n* = 18; **(C)** 4~7 days post-treatment, *n* = 14; **(D)** 8~21 days post-treatment, *n* = 3; and **(E)** ≥22 days post-treatment, *n* = 7.

The average z-spectra of the ischemic stroke lesions and CNAWM for the pre-treatment and post-treatment groups were demonstrated (Supplementary Figure S1). Figure 3 compared the average MTR_asym_ spectra of the ischemic stroke lesions and CNAWM pre-treatment and at several time points post-treatment. The CEST effect was clearly visible in the offset range of 1–4 ppm in the MTR_asym_ spectra. As reported before (25), the presence of the nuclear Overhauser enhancement (NOE) effects (29, 30) upfield from the water resonance caused a negative background for the asymmetry analysis of the z-spectra, leading to the negative CEST signals to be observed in the offset range of >4.5 ppm in the MTR_asym_ spectra. Notably, most CEST signals in the offset range of 2–5 ppm at pre-treatment were decreased in the ischemic stroke lesions, compared to CNAWM. The maximal change appeared at the offset of 3.5 ppm, where the amide protons of mobile proteins and peptides resonate. The APTW signal intensities of the ischemic stroke lesion increased gradually with post-treatment duration (negative values to positive values), and became even higher than those in the CNAWM at 8 ~ 21 days and ≥22 days post-treatment (0.65 ± 0.39% vs. 0.51 ± 0.19%, 0.82 ± 0.79% vs. 0.21 ± 0.35%, respectively, also see Table 2).

**Table 2:** Comparisons of APTW signal intensities (mean ± SD; % of the bulk water signal) for pre-treatment and post-treatment groups and *P*-values when comparing to pre-treatment.

	**Pre-treatment**	**≤ 96 h post-treatment**	**4 **~** 7 d post-treatment**	**8 **~** 21 d post-treatment**	**≥22 d post-treatment**
	**(*n* = 43)**	**(*n* = 18)**	**(*n* = 14)**	**(*n* = 3)**	**(*n* = 7)**
Lesion APTW	−1.01 ± 0.91	−0.45 ± 0.63	−0.23 ± 0.53	0.65 ± 0.39	0.82 ± 0.79
*P*-value	–	**0.004**	**< 0.001**	**< 0.001**	**< 0.001**
CNAWM APTW	0.36 ± 0.45	0.34 ± 0.42	0.19 ± 0.52	0.51 ± 0.19	0.21 ± 0.35
*P*-value		0.905	0.211	0.572	0.394
APTW contrast	−1.37 ± 0.87	−0.80 ± 0.68	−0.42 ± 0.38	0.14 ± 0.27	0.61 ± 0.59
*P*-value		**0.002**	**< 0.001**	**< 0.001**	**< 0.001**
NIHSS	6.0 ± 3.9	3.2 ± 1.6	3.1 ± 1.4	2.5 ± 0.7	1.4 ± 0.5
*P*-value	–	**0.006**	**0.002**	**< 0.001**	**< 0.001**

*CNAWM, contralateral normal-appearing white matter; NIHSS, National Institutes of Health stroke scale. Bold values means that P < 0.05*.

### Change of APTW Signals After Treatment

**Figure 4:**
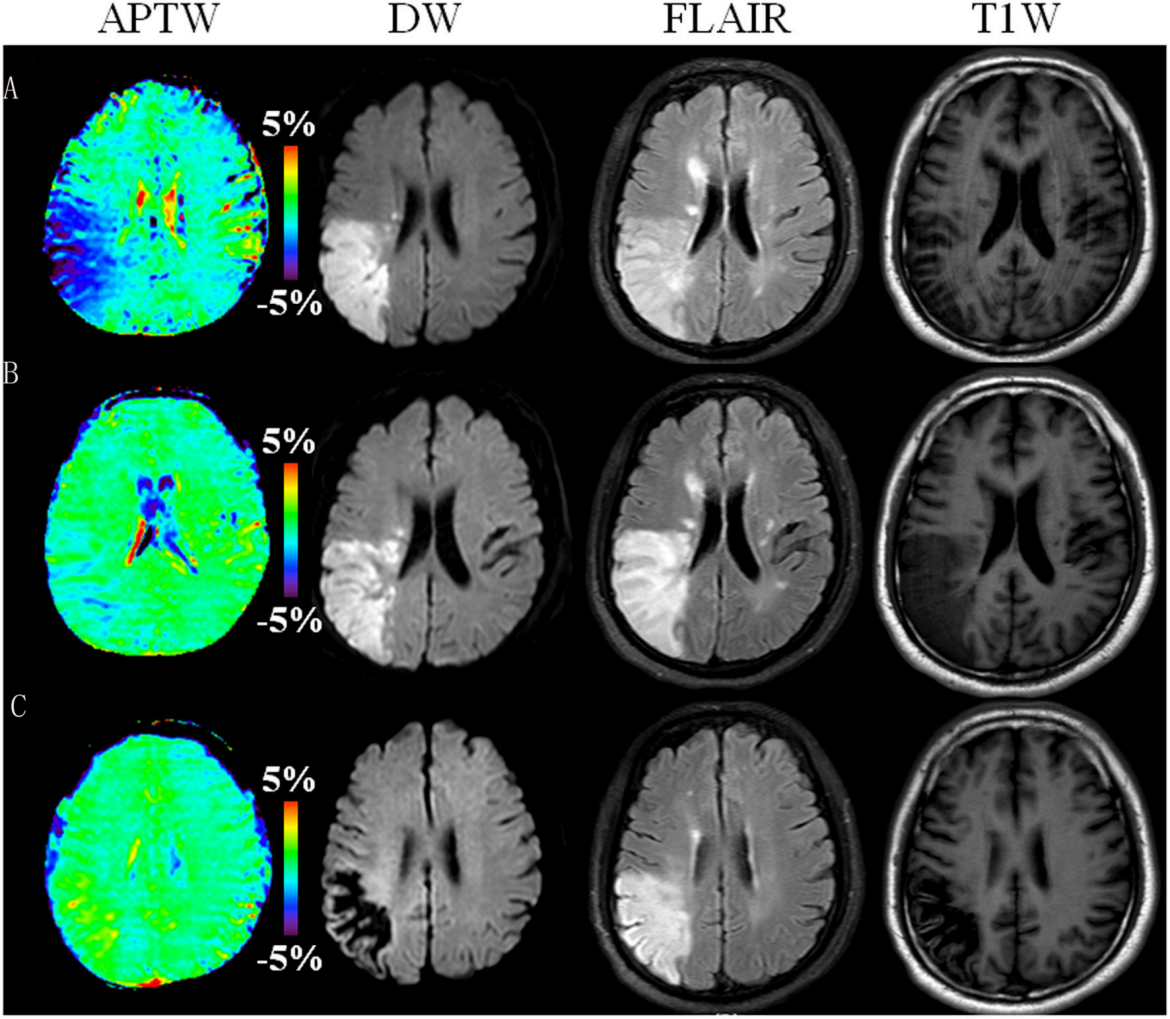
APTW, DW, FLAIR, and T_1_-weighted (T_1_W) images of a representative patient with ischemic stroke (male, 52 years old) at 1 day **(A)**, 6 days **(B)**, and 34 days **(C)** post-onset (or pre-treatment, 5 days, and 33 days post-treatment). DW images showed a hyperintensity in the lesion on the first and second scans, but hypointensity on the third scan. APTW images showed gradually increased signal intensity values in the corresponding lesion area. The NIHSS was 6, 3, and 2 at 1, 6, and 34 days post-onset, respectively. Note that the geometry and location of the slice was not positioned very well at the last time point.

Of 26 patients with follow-up scans after supportive treatment, 24 (92.3%) showed gradually increased APTW signal in the infarcted lesion with time, accompanied by an improvement in clinical symptoms. Figure 4 shows a typical case with effective treatment at 1, 6, and 34 days post-onset. At 1 day post-onset, DW images showed a hyperintensity in the lesion on the right frontal and parietal lobes, the APTW images showed a hypointensity in the same region, and the NIHSS was 6. At 6 days post-onset, DW images still showed the hyperintensity in the lesion, while the APTW images showed a considerable increase, and NIHSS decreased from 6 to 3. As for 34 days post-onset, DW images showed a hypointensity in the lesion. However, the APTW images had a further increased signal, which was even higher than that in the contralateral hemisphere, perhaps due to cystic liquefactive necrosis (31, 32). The NIHSS fell to 2, with further clinical improvement.

**Figure 5:**
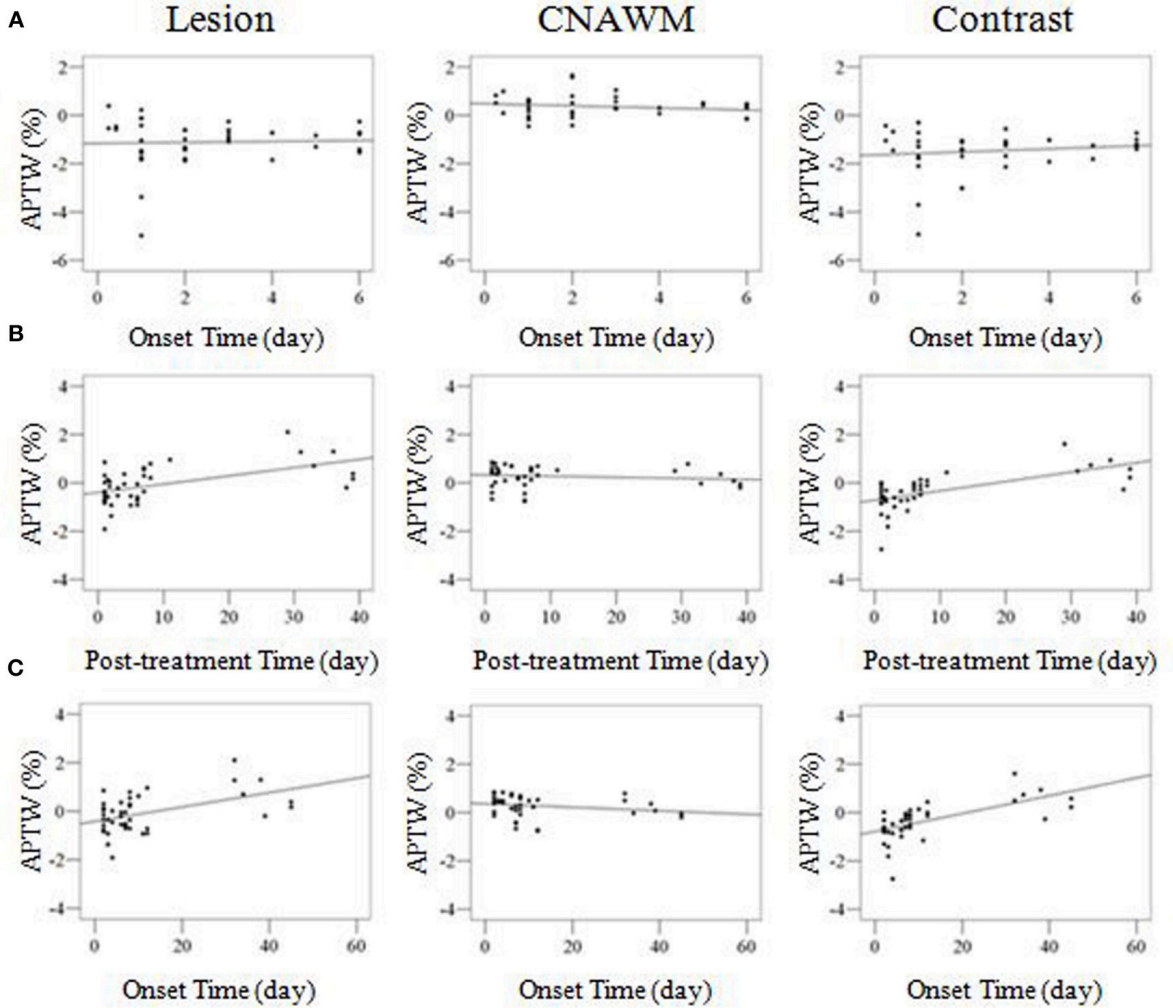
**(A)** Correlation analyses between APTW and onset time for the untreated patients, showing no significant correlations for lesion APTW (*r* = 0.043; *p* = 0.784), CNAWM APTW (*r* = −0.187; *p* = 0.229), and APTW contrast (*r* = 0.142; *p* = 0.362). **(B)** Correlation analyses between APTW signal intensities and post-treatment time for the treated patients. Lesion APTW (*r* = 0.537; *p* < 0.001), CNAWM APTW (*r* = −0.127; *p* = 0.422), and APTW contrast (*r* = 0.631; *p* < 0.001). **(C)** Correlation analyses between APTW signal intensities and onset time for the treated patients. Lesion APTW (*r* = 0.484; *p* = 0.001), CNAWM APTW (*r* = −0.211; *p* = 0.179), and APTW contrast (*r* = 0.625; *p* < 0.001). Both **(B,C)** show significant correlations for lesion APTW and APTW contrast, but no significant correlations for CNAWM APTW.

We quantitatively assessed the APTW signal changes before and after treatment. For the 24 patients with effective treatment, a significant increase in APTW values in the infarcted lesions was observed at all four time points post-treatment (Table 2). A significant NIHSS decrease was also seen at all time points post-treatment. In addition, we observed the significant APTW signal difference between the pre-treatment and post-treatment patients with the same post-onset time (Table 3). At two post-onset times of ≤96 h and 4–7 days, the APTW signal intensities were significantly higher in the post-treatment patients than in the pre-treatment patients, consistent with improved NIHSS in the post-treatment patients, while the CNAWM APTW showed no significant differences. The data for two later post-onset times of 8–21 days and ≥22 days were not analyzed because no untreated subjects had been recruited. This corresponded to significant correlations between lesion APTW signal intensities and post-treatment time (Figure 5B) or stroke onset time (Figure 5C) for the treated patients.

**Table 3:** Comparisons of APTW signal intensities (mean ± SD; % of the bulk water signal) for pre-treatment and post-treatment groups with different stroke onset times.

	**Onset time ≤96 h**			**Onset time 4 ~ 7 days**			**Onset time 8 **~** 21 days**	**Onset time ≥22 days**
	**Pre-treatment (*n* = 30)**	**Post-treatment (*n* = 12)**	***P*-value**	**Pre-treatment (*n* = 13)**	**Post-treatment (*n* = 11)**	***P*-value**	**Post-treatment (*n* = 12)**	**Post-treatment (*n* = 7)**
Lesion	−1.13 ± 1.05	−0.33 ± 0.61	**0.019**	−0.75 ± 0.45	−0.30 ± 0.34	**0.011**	−0.05 ± 0.69	0.82 ± 0.79
CNAWM	0.43 ± 0.50	0.46 ± 0.28	0.862	0.20 ± 0.26	0.19 ± 0.50	0.938	0.27 ± 0.57	0.21 ± 0.35
APTW contrast	−1.56 ± 1.01	−0.79 ± 0.51	**0.017**	−0.95 ± 0.46	−0.49 ± 0.32	**0.013**	−0.31 ± 0.57	0.61 ± 0.59
NIHSS	6.3 ± 4.2	3.2 ± 1.6	**0.002**	5.5 ± 3.7	3.1 ± 1.4	**0.048**	2.4 ± 0.8	1.6 ± 0.6

*CNAWM, contralateral normal-appearing white matter; NIHSS, National Institutes of Health stroke scale. Bold values means that P < 0.05*.

The remaining two patients (7.7%) showed further decreased APTW in the infarcted lesion on the second scan (the first scan after treatment), accompanied by clinical symptom aggravation. For example, the first case was scanned at 3 days post-onset, showing the DWI hyperintensity and APTW hypointensity (lesion APTW = −1.10%) on the right frontal lobe, with an NIHSS of 3. One day later (post-treatment), DW images still showed a hyperintensity in the lesion, while APTW images showed further decreased signal (lesion APTW = −1.91%), with an increase of the NIHSS to 6. The symptoms of this patient further worsened, and his NIHSS reached 8 at 11 days post-onset. These two patients whose treatment was ineffective were excluded from the quantitative analysis in Tables 2, 3.

## Discussion

Based on our results, baseline pre-treatment APTW signals correlated, inversely and significantly, with the baseline NIHSS, even though the infarction occurred in different regions of the brain. After treatment, most patients (over 90%) showed a gradually increasing APTW MRI intensity over time, accompanied by clinical symptom improvements and, therefore, an improvement in NIHSS; however, few patients showed a clearly decreasing APTW MRI intensity, accompanied by clinical symptom aggravation. APTW MRI is most likely sensitive to intracellular acidosis in stroke, including in animal models (33–36) and in patients (19–22), because of its sensitivity to pH changes. The increase in APTW MRI intensity indicates amelioration of intracellular acidosis, or relief of brain tissue ischemia and hypoxia. This explains the improvement in clinical symptoms. In contrast to this, the decrease in APTW MRI intensity indicates an aggravation of brain tissue ischemia and hypoxia, which would result in more serious clinical symptoms. It appears that APT imaging can provide unique information about post-treatment status and make the therapeutic effect visible. This also makes it possible to predict treatment effect by observing the APTW MRI signal changes after supportive treatment, especially the changes on the first scan after treatment. Increased APTW values may predict a good treatment effect, and decreased APTW values may predict a poor treatment effect at the very beginning of supportive treatment, which will help neurologists to make proper adjustment to treatment. Thus, the APTW signal may have the potential to serve as a new predictable imaging biomarker, additional to the MRI biomarkers listed in the review of Kidwell (37).

To explore the effect of supportive treatment on ischemia, we investigated longitudinal changes in APTW signal intensities based on post-treatment duration and onset time (Tables 2, 3, as well as Figure 5). For the patients with effective treatment, increased APTW values were observed with post-treatment duration, likely indicating the pH increase, which could be used to explain the improved symptoms and NIHSS improvement. For the onset times ≤96 h and 4–7 days, the APTW MRI signals of the treated patients were significantly higher than those of the untreated patients, which may have indicated a pH increase. Note that the change in APTW between these two untreated patient groups may reflect spontaneous recovery, which can be supported by the decrease NIHSS. The untreated patients with an onset time of 8–21 days and ≥22 days were not analyzed in this study, because there were no cases collected, which is not practical in the clinic. However, we could still see an increasing tendency in APTW signals after treatment in patients who had an onset time of 8–21 days. These results may indicate that the pH value of the infarction increased and cerebral alkalosis occurred at a late stage after treatment. This might be indicative of the influence of treatment, which would result in a pH increase for the relief of intracellular acidosis in the infarcted region.

The APTW signal quantified from MTR_asym_(3.5 ppm), as used in this study, was contaminated with the upfield NOE signal from mobile and semisolid proton types (29, 30). To obtain more pure APT signal, several alternative APTW imaging analysis or acquisition approaches have been proposed recently (38–42), such as Lorentzian-line-fit analysis and Bayesian model-based analysis (43). For example, it has been shown recently on stroke patients (44) that APTW imaging quantification using the extrapolated semi-solid MT model reference approach (41, 42) can achieve not only more pure APTW signals but also higher detection sensitivity, which may allow reliably delineation of ischemic penumbra tissues. However, these methods typically require a longer acquisition time, and their use for the routine practice requires further validation. Further, according to the theory (45), in addition to tissue pH, some other tissue factors (amide proton concentration, water proton concentration, and T_1_ of water) may affect the measured APTW signal. However, it is very important to realize that the contributions of tissue water content and T_1_ to APTW are mostly compensated in many diseases, as discussed in the previous papers (13, 46). Notably, in spite of its complexity, some recent numerical simulation studies (47, 48) have clearly demonstrated that the APT effect in tissue is actually not associated with water T_1_ at the saturation power of 2 μT used in this study. Consequently, the fact that the APTW signal increased during stroke recovery may suggest the amelioration of intracellular acidosis or even the occurrence of cerebral alkalosis, consistent with some early ^31^P MR spectroscopy studies (49, 50), but further validation is required.

In order to determine the robustness of the multiple small ROI selection method used, we further evaluated the mean APTW values and mean APTW contrasts across the infarct area drawn in the whole hyperintense brain regions on DW images (Supplementary Results). The lowest APTW seemed to provide more or similar information than the mean APTW of the whole lesion.

Some other limitations to this study should also be mentioned. First, we used 2D APT sequences in this study, and thus, only one slice was evaluated. To minimize possible error, we chose the central slice with the largest infarct size. In a planned future study, 3D APT sequences, as reported in the literature (51), would be used. Second, some other factors which may correlate with the treatment effect, such as the residual stenosis or occlusion of the affected vessel(s), the collateral flow, the degree of diffusion-perfusion mismatch, and the infarct volumes, were not analyzed in the present study. Third, the sample number was relatively small, and MRI scans had the very heterogeneous time points. Because of the sample size limitation, we did not statistically analyze the difference between the effective and ineffective (only two cases) treatment groups, although we could infer that a significant decrease in APTW signal could be found after treatment. Further analysis with more samples is needed in the future. Fourth, in this work, we focused on the recovery at sub-acute and early chronic stages post-stroke. A further study with patients at hyper-acute and acute stages will be performed in the near future. Finally, a further study should be performed to determine the utility of APTW signal as imaging biomarker to predict the final outcome of patients. The utility of APTW in the decision of thrombolysis and endovascular treatment of acute stroke patients, particularly in a multicenter trial, should be another interesting study to be performed in the future.

## Conclusion

APTW MRI is a promising non-invasive technique with which to characterize pH changes in the infarcted lesion in ischemic stroke patients. The increase in APTW signals in comparison to CNAWM may indicate an improvement in clinical symptoms, while a decrease in APTW signals may indicate a worsening of clinical symptoms. These findings would provide a new insight into the microenvironmental change in the ischemic stroke lesion with treatment. Therefore, APTW MRI can enable a visual assessment of the therapeutic effect because of its sensitivity to pH changes, and the APTW signal could serve as a useful surrogate biomarker with which to quantify the therapeutic effect of ischemic stroke treatments.

## Ethics Statement

This study was carried out in accordance with the recommendations of the human ethics committee of the Beijing Hospital with written informed consent from all subjects. All subjects gave written informed consent in accordance with the Declaration of Helsinki. The protocol was approved by the human ethics committee of the Beijing Hospital.

## Author Contributions

LY and CL: conducted the MRI data processing and statistical analyses, and drafted the initial manuscript; LY, YC, MC, XL, HC, TG, and CL: contributed to data collection and analyses; SJ, YZ, and JZ: assisted with data analysis and interpretation; CL: designed the study.

### Conflict of Interest Statement

The authors declare that the research was conducted in the absence of any commercial or financial relationships that could be construed as a potential conflict of interest.
