# Supplementary material for: Amide Proton Transfer MRI Signal as a Surrogate Biomarker of Ischemic Stroke Recovery in Patients With Supportive Treatment
Source: Front Neurol. 2019 Feb 22;10:104. doi: 10.3389/fneur.2019.00104 (PMC6395437; doi:10.3389/fneur.2019.00104)
Supplement: Supplementary file 1 [file Data_Sheet_1.PDF]

frontiers

Supplementary Material

Amide Proton Transfer-Weighted MRI Signal as a Surrogate Biomarker of Ischemic Stroke Recovery in Patients with Supportive Treatment

Lu Yu, MD, Yuhui Chen, MD, Min Chen, MD, Xiaojie Luo, MD, Shanshan Jiang, MD, Yi Zhang, PhD, Haibo Chen, MD, Tao Gong, MD, Jinyuan Zhou, PhD, and Chunmei Li, MD*

*** Correspondence:** Chunmei Li: bee9020@126.com

SUPPLEMENTARY RESULTS

For each infarct lesion, the whole infarct lesion was also manually drawn on the hyperintense brain regions on DW images by a radiologist who was blinded to patient outcome.The CNAWM region had the similar size and covered the similar region as the infarct lesion. Both mean APTW values and mean APTW contrast values of the whole lesion had significant correlations with the NIHSS at arrival ($r = -0.422$; $p = 0.005$; $r = -0.326$; $p = 0.033$, respectively). The correlation between pre-treatment ADC of the whole lesion and NIHSS at arrival was not significant ($r = 0.09$, $p = 0.568$).

Supplementary Table S1. Correlation analyses between whole lesion mean APTW signal intensities and onset time for the untreated patients, as well as between whole lesion APTW signal intensities and post-treatment time/onset time for the treated patients.

		Whole Lesion APTW	CNAWM	APTW Contrast
Onset time for untreated patients	r	0.31	-0.047	0.349
	p	**0.043**	0.764	**0.022**
Post-treatment time for treated patients	r	0.452	-0.028	0.514
	p	**0.003**	0.862	**<0.001**
Onset time for treated patients	r	0.392	-0.114	0.522
	p	**0.01**	0.473	**<0.001**

Supplementary Table S2. Comparisons of APTW signal intensities (mean ± SD; % of the bulk water signal) for pre-treatment and post-treatment groups when comparing to pre-treatment.

	Pre-treatment (n = 43)	≤ 96 h post-treatment (n = 18)	4~7 d post-treatment (n = 14)	8~21 d post-treatment (n = 3)	≥22 d post-treatment (n = 7)
Whole Lesion APTW	-0.41 ± 0.71	-0.18 ± 0.48	-0.008±0.60	0.56 ± 0.26	0.66 ± 0.55
P value		0.227	0.063	**0.025**	**<0.001**
CNAWM APTW	0.28 ± 0.53	0.36 ± 0.43	0.26±0.60	0.54 ± 0.38	0.36 ± 0.39
P value		0.526	0.904	0.394	0.697
APTW contrast	-0.69 ± 0.70	-0.55 ± 0.35	-0.26±0.58	0.01 ± 0.55	0.30 ± 0.55
P value		0.324	**0.048**	0.101	**0.001**
NIHSS	6.0 ± 3.9	3.2 ± 1.6	3.1±1.4	2.5 ± 0.7	1.4 ± 0.5
P value		**0.006**	**0.002**	**<0.001**	**<0.001**

Supplementary Table S3. Comparisons of APTW signal intensities (mean ± SD; % of the bulk water signal) for pre-treatment and post-treatment groups with different stroke onset times. There were no significant APTW signal differences between the pre-treatment and post-treatment patients with the same post-onset time.

	Onset time ≤ 96 hours			Onset time 4~7 days			Onset time 8~21 days	Onset time ≥ 22 days
	Pre-treatment (n = 30)	Post-treatment (n = 12)	P value	Pre-treatment (n = 13)	Post-treatment (n = 11)	P value	Post-treatment (n = 12)	Post-treatment (n = 7)
Whole Lesion	-0.54 ± 0.74	-0.14± 0.48	0.91	-0.10 ± 0.54	-0.16 ± 0.46	0.795	0.18 ± 0.72	0.65 ± 0.55
CNAWM	0.30±0.47	0.50±0.29	0.191	0.21 ± 0.64	0.26 ± 0.64	0.872	0.34 ± 0.58	0.36 ± 0.39
APTW contrast	-0.85±0.69	-0.64±0.38	0.343	-0.32 ± 0.60	-0.41 ± 0.43	0.658	-0.17 ± 0.54	0.30 ± 0.55
NIHSS	6.3 ±4.2	3.2 ±1.6	**0.002**	5.5 ±3.7	3.1 ±1.4	**0.048**	2.4 ±0.8	1.6 ±0.6

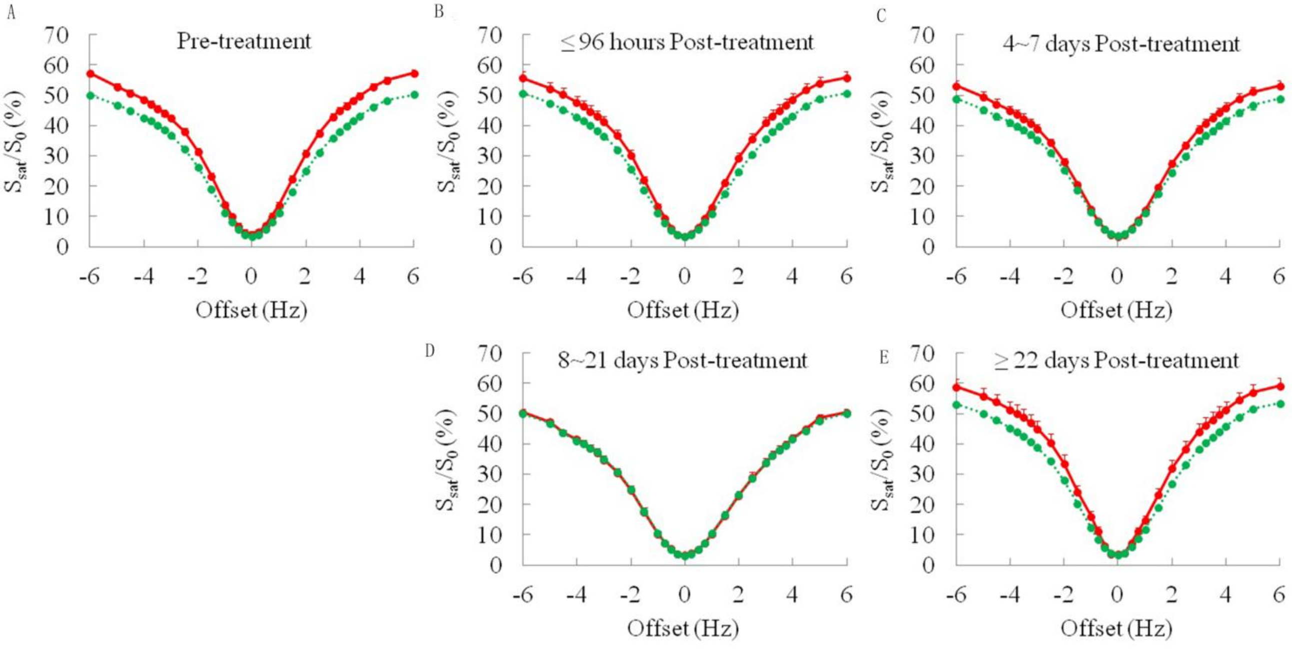

Supplementary Figure S1. The average z-spectra of the ischemic stroke lesions (red line) and CNAWM (green line) for the pre-treatment (A) and post-treatment (B-E) groups (mean ± standard error), corresponding to n = 43, 18, 14, 3, and 7, respectively.
